# Spatial Heterogeneity of Carbon Emissions and Its Influencing Factors in China: Evidence from 286 Prefecture-Level Cities

**DOI:** 10.3390/ijerph19031226

**Published:** 2022-01-22

**Authors:** Chen Li, Heng Li, Xionghe Qin

**Affiliations:** 1 School of Management, Shanghai University of Engineering Science, Shanghai 201620, China; 22150003@sues.edu.cn; 2 School of Economic and Management, Huainan Normal University, Huainan 232038, China; liheng968@163.com; 3 School of Urban and Regional Science, East China Normal University, Shanghai 200062, China

**Keywords:** carbon emissions, spatial heterogeneity, Theil index, 286 prefecture-level cities, China

## Abstract

In the face of the severe challenge of global warming, promoting low-carbon emission reductions is an important measure to cope with global climate change and achieve a green cycle of sustainable development. The purpose of this study was to reveal the spatial heterogeneity of carbon emissions and the influencing factors in 286 prefecture-level-and-above cities in China, and to provide an empirical basis for the formulation of low-carbon emission reduction policies in China. This study used a combination of comparative analysis, regional difference analysis, correlation analysis, principal component analysis, and stepwise regression analysis to analyze the spatial differences in carbon emissions and their influencing factors in 286 prefecture-level-and-above cities in China, and draws the following main conclusions: (1) From 2005 to 2015, regional differences in six sectors, including household carbon emissions, widened in the 286 prefecture-level-and-above cities in China, while regional differences in 14 sectors, including rural household carbon emissions, narrowed. (2) There were significant intra-group differences in urban household carbon emissions, and the contributions to intra-group differences in carbon emissions differed across the six sectors in the northeast, east, central, and west regions. (3) Although the total and average carbon emissions of each sector increased from 2005 to 2015, China’s carbon emission intensity was decreasing, and carbon productivity is increasing. (4) Carbon emissions per capita (CCE) were positively correlated with GRP per capita, industrial SO_2_ emissions per capita, and the proportion of employees in the secondary sector, and negatively correlated with population density and the proportion of employees in the tertiary sector. (5) Resident savings and consumption factors, pollution emission factors, and economic structure factors had a facilitating effect on CCE, while population density factors and economic growth factors have a weakening effect on CCE.

## 1. Introduction

Global warming has become a serious challenge that threatens the survival and sustainable development of mankind, and promoting low-carbon emission reductions is an important step in addressing global climate change and achieving green cycle sustainable development [1,2,3]. However, China has a long way to go in terms of low carbon emission reductions. The increase in carbon emissions mainly comes from the expansion of the economy [4,5,6,7], which in turn further stimulates the consumption of energy and the continuous growth of carbon emissions [8,9,10]. The China Urban Greenhouse Gas Emissions Dataset (2015) shows that Beijing and Shanghai, the two largest cities in China, have per capita carbon emissions of 7.33 tonnes per person and 11.46 tonnes per person, respectively, while Copenhagen, Paris, New York, Tokyo, and London have per capita carbon emissions of 2.87 tonnes per person, 3.50 tonnes per person, 7.96 tonnes per person, 4.97 tonnes per person, and 5.03 tonnes/capita. The two most-developed mega-cities in China have higher per capita carbon emissions than most world-renowned cities [11]. There are also significant regional differences in the intensity of carbon emissions, due to the different stages and regional economic development levels in China. Therefore, strengthening research on the spatial heterogeneity of carbon emissions in Chinese cities, measuring regional variations in carbon emissions by sector, and accurately portraying the spatial heterogeneity of carbon emissions, in terms of spatial and temporal evolution patterns and their influencing factors, are of reference value in promoting the coordinating the low-carbon development of China and improving the scientific and targeted nature of energy conservation and emission reduction policies.

Based on the above background, the study of carbon emissions has rapidly become a hot topic in academic circles, and scholars have conducted research on measurement methods, influencing factors, and the spatial heterogeneity of carbon emissions, among which the spatial heterogeneity of carbon emissions, as an important element of low-carbon emission reductions, is one of the key concerns of scholars.

(1) In terms of research topics, Wang, Su, and Zhao quantitatively identified the evolutionary pathways of urban carbon emissions in China [12]. Li, Ma, and Wei explored the characteristics and variability of energy carbon emission efficiency in different regions in China [13]. Qiu and Xu conducted an empirical study on the spatial and temporal differences in carbon emissions of 13 representative cities in eight urban agglomerations in China [14]. Wang et al. used a super-efficient SBM model to measure urban carbon emission performance [15]. Wu analyzed the time-series evolution and spatial variation of carbon emissions across cities in Guangdong province [16]. Xu studied the per capita carbon emissions in Jiangsu Province from 2003 to 2018 [17]. Regarding the analysis of spatial heterogeneity of sectorial carbon emissions, scholars have measured and analyzed the spatial heterogeneity of carbon emissions from the agriculture sector [18,19,20], services sector [21], manufacturing sector [22], transportation sector [23,24], and food consumption sector [25] in Chinese cities, respectively. Most of the existing studies analyzed the spatial heterogeneity of national, regional, and, especially, sectorial carbon emissions, failing to combine the three and, thus, lacking in relativity and comprehensiveness. At present, most studies are limited by the availability of data and focus on the spatial heterogeneity of carbon emissions at large scales, such as national, regional, and provincial scales, while most studies at the city scale focus on the time series analysis of carbon emissions of individual cities, especially those with more complete data for municipalities directly under the central government, but they rarely involve regional differences in the spatial heterogeneity of urban carbon emissions at the national level. In general, the existing literature on the spatial heterogeneity of carbon emissions mainly focuses on the provincial spatial scale, while there are few studies at an urban scale.

(2) In terms of research methods, the main methods for measuring carbon emissions are the emission factor method, input–output method, life-cycle method, material balance method, actual measurement method, and modeling method. Specifically, Schipper used the factor decomposition method to analyze the carbon emission intensity of 13 IEA countries [26]. Casler used the modeling method to structurally analyze carbon emissions in the US [27]. Chang et al. studied Taiwan’s industrial carbon emissions and their structural decomposition based on the input–output method [28]. Guo used a structural decomposition analysis on the basis of the input–output method to decompose the growth of carbon dioxide in China [29]. Scholars have also used the Theil index, Dagum Gini coefficient, and other methods to measure variables such as total carbon emissions and carbon emission efficiency, in order to examine the spatial heterogeneity of carbon emissions in China [30,31,32,33,34]. On this basis, some studies used spatial analysis methods such as Moran’s I index and generalized spatial model, to reveal the spatial effects of carbon emissions in China [35,36,37]. The above studies focused on the influence of industrial structure on carbon emissions, but did not consider the intensity of carbon emissions and their heterogeneity in different sectors and regions.

(3) In terms of research data, given the availability of data, scholars mostly use the technical classification method recommended by the IPCC to estimate carbon emissions, and most Chinese scholars account for carbon emissions based on officially published energy consumption statistics. Although they used night scene lighting data to estimate carbon emissions across the country, and compared the spatial heterogeneity of carbon emissions in different spatial units, such as regions, provinces, and cities [38,39,40,41,42], their data are still based on experimental estimation, and the accuracy of this estimation needs to be improved. Due to the differences in the caliber of energy statistics among provinces, it is complicated to perform carbon emission accounting, resulting in a lack of research on the heterogeneity of urban carbon emissions and influencing factors based on the national level. Although scholars have conducted spatio-temporal simulations of carbon emissions in Chinese cities, based on satellite nighttime lighting data [43,44], and used urban remote sensing simulations to invert carbon emissions data and conduct evolutionary simulation analyses of carbon emissions in Chinese cities, studies on the spatial heterogeneity of carbon emissions at the city scale have mainly used simulated data, and their data accuracy needs to be improved.

(4) In terms of influencing factors, researchers have explored the influencing factors of carbon emissions using the IPAT model and its extension STIRPAT model, Kaya’s constant equation, and the LMDI exponential decomposition method [45,46,47]. Padilla analyzed the impact of national income differences on CO_2_ emission differences [48]. Sather used the coefficient of variation, Gini coefficient, and Theil index to measure the spatial differences in CO_2_ emissions between the eastern, central, and western regions of China [49]. Shao used the Tapio decoupling model to analyze the impact of economic growth patterns on regional carbon emissions in six central provinces of China [50,51,52]. Although research has initially been specific to the issue of factors influencing the spatial heterogeneity of carbon emissions, extensive studies have been conducted in the academic community, but no consistent conclusions have been reached, and the analysis of the factors influencing spatial heterogeneity still needs to be further explored.

In summary, many scholars have analyzed the spatial heterogeneity of carbon emissions in China at national, regional, and provincial spatial scales, and have achieved rich results. However, due to data limitations, these studies are mainly based on provincial data, and few scholars have examined the spatial heterogeneity of carbon emissions in China using city unit data. In fact, the spatial heterogeneity of carbon emissions across different urban units is even more pronounced, and the study of urban data is crucial for capturing regional heterogeneity and formulating reasonable and effective carbon emission reduction policies. Although some scholars have measured carbon emissions in typical cities based on carbon emission data, their studies were limited to smaller spatial areas and could not grasp the overall pattern of carbon emissions in China from a comprehensive and overall perspective.

Unlike previous studies, this study adopted the Theil index, principal component analysis, and stepwise regression analysis models to comprehensively examine the spatial heterogeneity of carbon emissions in China and its influencing factors, based on carbon emission data from 286 cities at the prefectural level and above in China. The contributions of this paper are mainly reflected in: first, in terms of sample data, the data of the Chinese urban greenhouse gas emission dataset were used in this paper, which draws on the collective wisdom of 137 scientists in China, and lays a solid research foundation for a comprehensive examination of the spatial heterogeneity of carbon emissions in China; second, in terms of spatial scale, the Theil index was used to decompose the eastern region, the central region, the western region, and the northeastern region, at the spatial scale. Finally, the Theil index was used to decompose the structure of the intrinsic differences in carbon emissions of 20 sectors in the cities of four regions, including the eastern, central, western, and northeastern regions, and to compare the intra-group and inter-group differences in carbon emissions of each sector. This study will provide a scientific basis for the formulation of the ‘Carbon Summit’ action plan for 2030.

Our research objects were 286 cities in four regions of eastern, western, central, and northeastern China. There are significant spatial differences in regional features, such as the population density, economic activity, and weather conditions among the four regions. The spatial difference of regional economic development level presents a significant step-by-step distribution characteristic. The economic development level decreased from coastal areas to inland areas, with the highest in the eastern region, the second in the central region, and the lowest in the west and northeast regions. The geographical distribution of China’s urban population is uneven, with a densely populated eastern area and sparsely populated western area. This spatial distribution pattern is separated by the ‘Hu Huanyong Line’, an imaginary line that divides China into two parts with roughly equal populations, from Heihe (a northern Chinese city bordering Russia) to Tengchong (a southwestern city bordering China with Myanmar) [53,54]. During the study period, the overall migration of China’s population mainly flowed from the northeast and west regions to the central and eastern regions. In terms of the weather conditions, the southeastern areas at low latitudes have higher temperatures and more precipitation throughout the year, while the northwestern areas are hot and rainy in summer, and cold and dry in winter. The spatial differences of these factors may be somewhat related to the spatial heterogeneity of carbon emissions in China.

The purpose of this study was to reveal the spatial and temporal differences between sectors in China and to identify the main drivers of carbon emissions in China. The organizational framework of this paper is as follows: Part I is an introduction; Part II is an introduction to the methodology and data sources; Part III is an analysis of regional differences and a comparative analysis of carbon emissions by sector; Part IV is a correlation analysis, principal component analysis and stepwise regression analysis of the factors influencing carbon emissions in 286 prefecture-level-and-above cities in China; Part V has a discussion; Part VI get conclusions; and Part VII has policy recommendations.

## 2. Methods

### 2.1. Research Methods

#### 2.1.1. Spatial Heterogeneity Analysis

The Theil index examines inequality and disparity from the concepts of information quantity and entropy, and it decomposes overall disparity into disparity between parts and disparity within parts, which has wide applications for analyzing and decomposing disparity and inequality [55,56,57].The composite entropy index examines the variability among individuals from the concepts of information quantity and entropy, which is the expected value of information quantity, i.e., the expected information quantity [58,59]. The closer the individuals are to each other, the smaller the composite entropy index will be.

(1) $$GE=\left\{ \begin{array}{l} \sum_{i=1}^{n}p_{i}[{\left(y_{i}/u\right)}^{c}-1],c\neq 0,1\\ \sum_{i=1}^{n}p_{i}(y_{i}/u)lg(y_{i}/u),c=1\\ \sum_{i=1}^{n}p_{i}lg(y_{i}/u),c=0 \end{array}\right.$$

In Equation (1), the parameter c is used to determine the sensitivity of the exponential change. In general, when c < 2, the exponential change it determines is sensitive. When c = 0.1, it is the well-known Theil’s index.

Due to its property of dividing overall differences into within-group differences and between-group differences, the Thayer index is widely used in empirical studies of overall spatial heterogeneity, as well as inter-spatial heterogeneity. The calculation formula is

(2) $$Theil=\sum_{i=1}^{n}T_{i}ln(nT_{i})=T_{WR}+T_{BR}$$

If the area under study is divided into groups according to certain methods, the Theil index can be further decomposed into intra-group differences and inter-group differences.

(3) $$T_{WR}=\sum_{i=1}^{n_{db}}T_{i}ln(n_{db}\frac{T_{i}}{T_{db}})+\sum_{i=1}^{n_{d}}T_{i}ln(n_{d}\frac{T_{i}}{T_{d}})+\sum_{i=1}^{n_{z}}T_{i}ln(n_{z}\frac{T_{i}}{T_{z}})+\sum_{i=1}^{n_{x}}T_{i}ln(n_{x}\frac{T_{i}}{T_{x}})$$

(4) $$T_{BR}=T_{db}ln(T_{db}\frac{n}{n_{db}})+T_{d}ln(T_{d}\frac{n}{n_{d}})+T_{z}ln(T_{z}\frac{n}{n_{z}})+T_{x}ln(T_{x}\frac{n}{n_{x}})$$

In Equations (3)–(5), Theil is Theil index; n is the number of cities in the sample region; T_WR_ is the differences within the four regional groups of northeast, east, central, and west regions; T_BR_ is the differences between the four regional groups; n_db_, n_d_, n_z_, and n_x_ are the number of cities within the eastern, eastern, central, and western regions, respectively; Ti is the carbon emission index of region in and the national average ratio; T_db_, T_d_, T_z_, and T_x_ are the ratios of carbon emission indexes of the northeast, east, central, and west regions to the national average, respectively.

A total of 286 cities at the prefecture level and above are included in our study. The northeast region includes 34 cities in Liaoning, Jilin, and Heilongjiang; the east region includes 87 cities in Beijing, Tianjin, Hebei, Shanghai, Jiangsu, Zhejiang, Shandong, Fujian, Guangdong, and Hainan; the central region includes 80 cities in Shanxi, Henan, Anhui, Hubei, Hunan, and Jiangxi; and the west region includes 85 cities in Inner Mongolia, Chongqing, Sichuan, Guangxi, Guizhou, Yunnan, Shaanxi, Gansu, Ningxia, Tibet, Qinghai, and Xinjiang.

#### 2.1.2. Correlation Analysis

The scatter plot is the most visual method used to express correlation analysis. The correlation coefficient is a collective term for a class of indicators that measure the correlation between variables [60,61,62].

The common correlations are linear correlation, curvilinear correlation, positive correlation, and negative correlation. The Pearson correlation coefficient, also known as the product–difference correlation coefficient, is a common metric for quantitatively describing the degree of linear correlation [63,64,65]. The formula for measuring the Pearson correlation coefficient is.

(5) $$r=\frac{\sum \left(x_{i}-\bar{x})(y_{i}-\bar{y}\right)}{\sqrt{{\left(x_{i}-\bar{x}\right)}^{2}}\sqrt{{\left(y_{i}-\bar{y}\right)}^{2}}}$$

In Equation (5), x_i_ and y_i_ are the variables, $\bar{x}$ and $\bar{y}$ are the means of variables x_i_ and y_i_, and r is the correlation coefficient. The maximum correlation coefficient is 1. The closer the absolute value of the correlation coefficient is to 1, the stronger the correlation between the variables. Our study will measure the correlation coefficient between carbon emissions per capita and its influencing variables using correlation coefficients, and visually express the relationship between them by means of scatter plots.

#### 2.1.3. Principal Component Analysis

Principal component analysis is a multivariate statistical method for examining correlations among multiple variables, and its application can be reduced to the purposes of data compression and data interpretation [66,67,68,69,70]. Specifically, it examines how the internal structure among multiple variables can be explained by a few principal components. In other words, numerous indicators are substituted for the original indicators by means of dimensionality reduction, but are able to reflect the main content of the source indicators. Usually, N indicators are linearly combined and downscaled into a new composite indicator.

Often, principal component analysis is more of an intermediate means to an end than an end in itself, and it is often used as an intermediate step in many studies that continue with other multivariate statistical methods, after condensing the data in order to address the actual problem [71,72,73,74]. In our study, the principal component analysis is followed by a multiple regression analysis to explain the main influencing factors of carbon emissions.

A common method of principal component analysis is to express the variance using F_1_. The larger the variance, the more information is expressed by F_1_, so the maximum value of variance in the linear combination of F_1_ is the first factor. If F_1_ is not sufficient to represent the information of the original N indicators, then F_2_ is considered as the second factor, and so on [75,76]. The mathematical model of factor analysis is as follows:

(6) $$\left\{ \begin{array}{l} F_{1}=a_{11}ZX_{1}+a_{21}ZX_{2}+\cdots +a_{p1}ZX_{p}\\ F_{2}=a_{12}ZX_{1}+a_{22}ZX_{2}+\cdots +a_{p2}ZX_{p}\\ \cdots \cdots \\ F_{p}=a_{1m}ZX_{1}+a_{2m}ZX_{2}+\cdots +a_{pm}ZX_{p} \end{array}\right.$$

In Equation (3), a_1i_, a_2i_, …, a_pi_ (i = 1, 2, …, m) are the eigenvectors corresponding to the eigenvalues of the covariance matrix of X, and ZX_1_, ZX_2_, …, ZX_P_ are the standardized values of the original variables. Factor analysis was performed on the standardized indexes using SPSS statistical analysis software, and the factor principal factors were calculated by KMO and Bartlett’s sphericity tests, rotated using the maximum variance method, and finally the factor composite scores were determined.

#### 2.1.4. Regression Analysis

Correlation analysis considers only the correlation between variables and does not reveal their causal relationship, while regression analysis is concerned with the causal relationship between variables. Regression analysis is a statistical method that deals with the linear causal relationship between a dependent variable and one or more independent variables. Multiple linear regression models are suitable for analyzing the relationship between a dependent variable and multiple independent variables [60]. Suppose a regression model consists of p − 1 independent variables, i.e., x_1_, x_2_, …, x_p−1_, then the regression model can be expressed as:

(7) $$y_{i}=\beta_{0}+\beta_{1}x_{i1}+\beta_{2}x_{i2}+L+\beta_{k}x_{ik}+L+\beta_{\left(p-1\right)}x_{i(p-2)}+\varepsilon_{i}$$

In Equation (7), y_i_ denotes the value of individual i (i = 1,2, …, n) in the dependent variable, β_0_ is the overall parameter of the intercept, 1, 2, …, k, …, p − 1 are the overall parameters of the slope. Since this regression model contains multiple independent variables, it is called a multiple regression model. In social science research, we always try to be sure that the model is set up correctly, but sometimes the problem of including irrelevant independent variables in the model may arise. The problem of incorporating irrelevant variables may increase the problem of multi-collinearity and, thus, diminish the validity of the model estimates. Our approach is to use stepwise regression analysis to solve the multi-collinearity problem, making our model estimates more valid and explanatory.

### 2.2. Data Sources

The carbon emission data of 286 cities at prefecture level and above in China were collected from the China City Greenhouse Gases Emission Dataset (2005) and China City Greenhouse Gases Emission Dataset (2015) published by the China Environment Publishing Group and authored by the China Urban Greenhouse Gas Working Group [77]. Data on the influencing factor variables of carbon emissions were collected from China Urban Statistical Yearbook 2016 [78] and China Urban Construction Statistical Yearbook 2015 [79].

Using the quartile method, the per capita carbon emissions (CCE) and total carbon emissions (TCE) of 286 cities at the prefecture level and above in China were divided into four quartiles.

**Figure 1:**
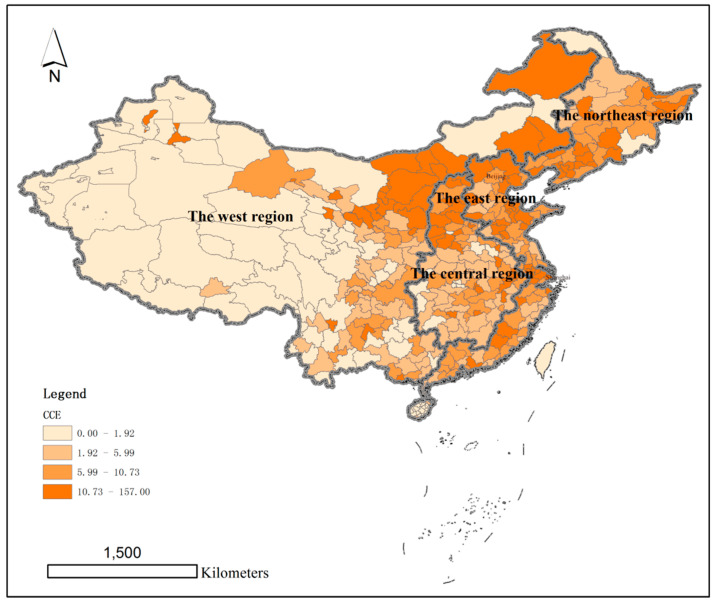
Capita carbon emissions in prefecture-level-and-above cities in China in 2015 (t/person).

The cities with per capita carbon emissions in the first quartile accounted for 10 cities, those with per capita carbon emissions in the second quartile accounted for 90 cities, those with per capita carbon emissions in the third quartile accounted for 93 cities, and those with per capita carbon emissions in the fourth quartile accounted for 93 cities. The highest value of carbon emission per capita is 157.01 tonnes per person (Karamay) and the lowest value is 1.11 tonnes per person (Beijing). There are 54 cities with per capita carbon emissions higher than the average value of 11.59 tonnes per person, accounting for 18.89%. The number of cities with per capita carbon emissions lower than the average value of 11.59 tonnes per person is 232, accounting for 81.11% (Figure 1).

**Figure 2:**
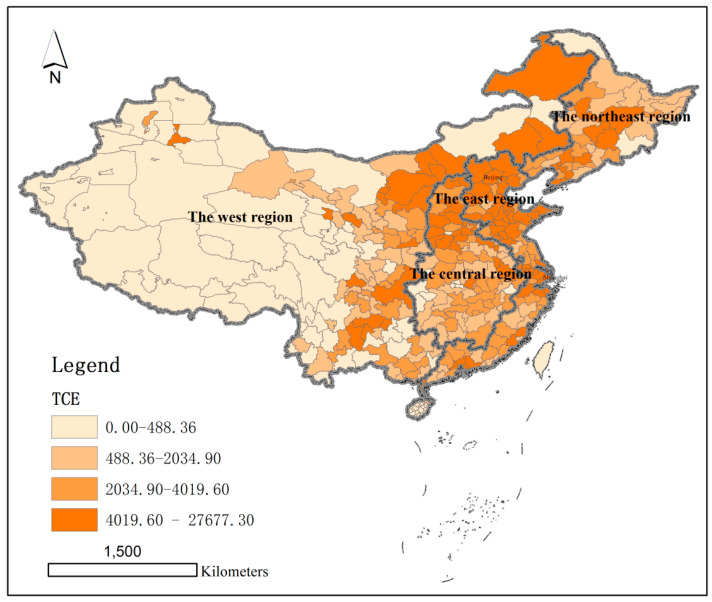
Total carbon emissions in prefecture-level-and-above cities in China in 2015(10^4^ tonnes).

The cities with total carbon emissions in the first quartile numbered 10, the cities with total carbon emissions in the second quartile numbered 91, the cities with total carbon emissions in the third quartile numbered 93, and the cities with total carbon emissions in the fourth quartile numbered 92. The highest value of total carbon emission is 276,773,200 tonnes (Shanghai) and the lowest value is 2,422,300 tonnes (Lhasa). There are 97 cities with total carbon emissions above the average value of 39.5042 million tonnes, accounting for 33.92%. There are 189 cities with total carbon emissions lower than the average value of 39.5042 million tonnes, accounting for 66.08% (Figure 2).

## 3. Analysis of Regional Differences in Carbon Emissions

### 3.1. Decomposition of Regional Differences in Carbon Emissions

#### 3.1.1. Within-Group Differences, between Group Differences, and Total Differences

From the measurement of spatial heterogeneity of carbon emissions in 20 sectors in China, the following information was derived (Table 1).

First, regional differences widened for six sectors: urban household carbon emissions, railway carbon emissions, aviation carbon emissions, carbon emissions per unit of GDP, carbon emissions per unit of GDP in the primary industry, and carbon emissions per unit of GDP in the secondary industry, while for rural household carbon emissions, agricultural carbon emissions, industrial carbon emissions, service carbon emissions, road carbon emissions, water navigation carbon emissions, transportation carbon emissions, direct carbon emissions, indirect carbon emissions, total carbon emissions, per capita carbon emissions, carbon emissions per unit of land area, carbon emissions per unit of GDP in the tertiary sector, and carbon productivity they narrowed; the regional differences in 14 sectors narrowed.

Second, the main reason for the widening of regional differences in six sectors is that the intra-group differences in carbon emissions have increased more than the inter-group differences. For example, the intra-group variance coefficient for urban household carbon emissions expanded from 0.485 in 2005, to 0.685 in 2015, an increase of 20.00% in the intra-group variance and −3.50% in the inter-group variance. In other words, the within-group variation in urban household carbon emissions is higher than the between-group variation, leading to a widening of the total variation in urban household carbon emissions.

Third, there are two main reasons for the narrowing of regional differences across the 14 sectors: one reason is that both intra-group and inter-group differences in carbon emissions across the 14 sectors appear to have narrowed, leading to a narrowing of the total difference. For example, the increase in intra-group and inter-group differences in carbon emissions for rural households was −3.80% and −7.50%, respectively, while the increase in the total difference was −11.30%. Second, the sum of the increase in intra-group and inter-group differences in carbon emissions for 14 sectors was negative. For example, the increase in the intra-group variance of carbon emissions in the service sector was −9.805, while the increase in the inter-group variance was 2.30%, resulting in the total variance of carbon emissions in the service sector remaining negative and the total variance showing a narrowing trend.

A preliminary analysis of the main reasons for the changes in spatial heterogeneity of carbon emissions in 20 sectors: (1) Regional differences in China’s carbon emissions are the result of the interaction of various forces, including the forces of urbanization, industrialization, policy reform, and economic development, etc. The way towards modernization of China’s low-carbon emission reduction involves from the intertwining and interaction of these forces. (2) The coefficients of variation in carbon emissions from urban households, railway carbon emissions, aviation carbon emissions, and carbon emissions per unit of GDP from secondary industries have become larger, precisely reflecting the process of industrialization and the rapid development of modern China. The years 2005–2015 saw a period of rapid urbanization in China, with China’s urbanization rate rising sharply from 42.99% in 2005, to 56.10% in 2015. During this period, China’s urban population increased sharply, from 562.12 million in 2005, to 771.16 million, a cumulative increase of 209.04 million in the urban population, which means a large influx of rural population into the cities. As such, they need a large amount of consumption and generate a large amount of carbon emissions, which invariably brings about changes in regional differences in carbon emissions of urban households. Similarly, in the process of rapid modernization, China has invested tremendously in infrastructure, with social fixed asset investment amounting to RMB 8877.36 billion in 2005 and increasing to RMB 5619.98 billion in 2015, an expansion of 6.33 times in the last 11 years. Obviously, the infrastructure construction invested in with a huge amount of capital has brought about an increase in energy consumption, and to some extent, a change in the spatial heterogeneity of carbon emissions in railways, aviation, and other sectors.

#### 3.1.2. Decomposition of within Group Differences in Four Regions of China

A further analysis of the spatial heterogeneity of carbon emissions in 20 sectors in China revealed the following intra-group differences among the northeastern, eastern, central, and western regions of China (Table 2):

First, the within-group differences in six sectors, namely urban household carbon emissions, railway carbon emissions, aviation carbon emissions, carbon emissions per unit of GDP, carbon emissions per unit of GDP in the primary industry, and carbon emissions per unit of GDP in the secondary industry, are significant, as shown by the different contributions of the within-group differences in carbon emissions in the six sectors in the northeast, eastern, central, and western regions. In terms of urban household carbon emissions, the increase in intra-group variation in the western region was significantly higher than that in the other three regions. In the railway sector, the within-group differences in the central and western regions widened slightly, while the within-group differences in the northeastern region showed a narrowing trend. In the aviation sector, the highest contribution to intra-group variation in carbon emissions was made by the western region, where the intra-group variation increased by 21.70%, while the increase in the eastern region and the eastern region was 4.90% and 2.60%, respectively; significantly lower than that of the western region. In terms of carbon emissions per unit of GDP in the secondary sector, the within-group difference in carbon emissions widened by more than 5.00% in both the eastern and central regions, while the within-group difference increased or decreased by less than 1.00% in both the eastern and western regions.

Second, within-group differences in carbon emissions from rural households, carbon emissions from water navigation, indirect carbon emissions, and carbon emissions per unit of GDP from the tertiary sector were significant across the 14 sectors. For rural household carbon emissions, the intra-group variation in the eastern region showed a widening trend, with the intra-group variation coefficient increasing by 25.60% from 2005 to 2015, while the intra-group variation in the western region showed a narrowing trend, with the intra-group variation increasing by −33.20%. In terms of carbon emissions from water navigation, the intra-group variation coefficients of the northeast, east, central and west regions increased by 9.70%, −24.80%, 11.30%, and 4.30%, respectively. In terms of indirect carbon emissions, the intra-group differences in the northeastern and eastern regions showed a significant reduction, while the intra-group differences in the central and western regions fluctuated by −2.10% and 0.60%, respectively. In terms of carbon emissions per unit of GDP in the tertiary sector, the within-group variation in the western region showed the largest change, with an increase of −23.10%, while the changes in the northeastern and eastern regions were smaller, at −0.90% and 1.60%, respectively.

To a large extent, the spatial heterogeneity of carbon emissions across the 20 sectors in China’s four regions was brought about by the varying magnitude of intra-group differences. The direct cause of the spatial heterogeneity of carbon emissions across the 20 sectors in China and its explanation requires a visual analysis of the carbon emissions data for each sector. Next, we will compare carbon emissions across the 20 sectors in the four regions. Prior to the comparative analysis, we group the 20 sectors into seven categories: carbon emissions from urban and rural households, carbon emissions from agriculture, industry and services, carbon emissions from roads, railways, water transport and aviation, carbon emissions per capita and carbon emissions per land area, direct and indirect carbon emissions, carbon emissions per unit of GDP, and carbon productivity.

### 3.2. Decomposition of Carbon Emissions

#### 3.2.1. Carbon Emissions from Urban and Rural Households

Looking at the total carbon emissions of urban and rural households, it is very surprising to find that the carbon emissions of rural households in China are higher than those of urban households. The carbon emissions ratio of urban households to rural household was 0.68 in 2005, rising to 0.71 in 2015, i.e., the carbon emissions of rural households are higher than those of urban households. The total carbon emissions of both urban and rural households showed a declining trend, with urban household carbon emissions in China’s 286 prefecture-level-and-above cities amounting to 126,499,400 tonnes in 2005, falling to 98,665,600 tonnes in 2015, a decline of 22%. The carbon emissions of rural households in China’s 286 prefecture-level-and-above cities were 187,145,400 tonnes in 2005, declining to 139,537,200 tonnes in 2015, a decline of 25.44%. In terms of the average amount of carbon emissions of urban and rural households, it is still the case that the per capita carbon emissions of rural households in China are higher than those of urban households. The average carbon emissions of households in China’s 286 prefecture-level- and-above cities was 654,400 tonnes in 2005, and the average dropped to 487,900 tonnes in 2015, a decrease of 25.44% (Table 3).

In terms of the average carbon emissions of urban and rural households in the four regions, although urban households in eastern China had the largest average carbon emissions, they have also seen the largest reduction in carbon emissions, from 638,200 tonnes to 407,300 tonnes, between 2005 and 2015, a reduction of 36.17%. Although rural households in northeast China had the smallest average carbon emissions, they also had the largest increase in carbon emissions. The average carbon emissions of rural households in Northeast China rose from 202,100 tonnes in 2005, to 336,100 tonnes in 2015, an increase of 66.31%. In addition, urban households in western China and rural households in central China also saw a small increase in carbon emissions.

#### 3.2.2. Carbon Emissions from Agriculture, Industry, and Services

From 2005 to 2015, China’s carbon emissions from the three major sectors of agriculture, industry, and services all increased significantly. In terms of the total carbon emissions from the three major sectors, in 2005, the total carbon emissions from the agricultural sector, industrial sector, and service sector in 286 prefecture-level-and-above cities in China were 73,116,900 tonnes, 54,914,300 tonnes, and 63,236,400 tonnes, respectively, while in 2015, the total carbon emissions of the agricultural sector, industrial sector, and service sector were 95,211,900 tonnes, 88,753,800 tonnes, and 284,754,600 tonnes, respectively; with the total carbon emissions of the three major sectors increasing by 1.30 times, 1.62 times, and 4.50 times, respectively. In terms of the average amount of carbon emissions from the three major sectors, in 2005, the average carbon emissions from the agricultural sector, industrial sector, and service sector in 286 prefecture-level-and-above cities in China were 255,700 tonnes, 19,199,400 tonnes and 221,100 tonnes respectively; in 2015, the average carbon emissions from the agricultural sector, industrial sector and service sector were 332,900 tonnes, 31,033,700 tonnes, and 995,600 tonnes, respectively.

In terms of the average carbon emissions of the four major regions in China, the average carbon emissions of the agricultural sector in the northeastern, eastern, central, and western regions of China were 289,800 tonnes, 338,000 tonnes, 240,800 tonnes, and 171,700 tonnes in 2005, respectively. The average carbon emissions of the agricultural sector in the northeastern, eastern, central, and western regions were 429,900 tonnes, 348,400 tonnes, 346,500 tonnes, and 265,400 tonnes in 2015, respectively. In addition, from 2005 to 2015, the average carbon emissions of both industry and services in the four major regions of China increased significantly, with the fastest growth being in the services sector in the northeast, where the average carbon emissions expanded by 7.65 times, and with the slowest growth being in the agriculture sector in the east, where the average carbon emissions expanded by 1.03 times (Table 4).

#### 3.2.3. Carbon Emissions from Road, Railway, Waterborne Navigation, and Aviation

The period 2005–2015 was one of the most intensive periods in China for the construction of ‘iron and public infrastructure’, i.e., railway, highway, airport, water conservancy, and other major infrastructure. In terms of total carbon emissions from the road, railway, water transport, and aviation sectors, the total carbon emissions from 286 cities at prefecture level and above in China were 298,542,000 tonnes, 13,446,700 tonnes, 23,083,800 tonnes, and 19,733,400 tonnes in 2005, respectively, while the total carbon emissions from road, railway, water transport, and aviation were 63,964,300 tonnes, 7,637,400 tonnes 40,980,000 tonnes, and 55,426,200 tonnes in 2015, respectively, with the total carbon emissions of the four major industries increasing by 2.14 times, 0.57 times, 1.78 times, and 2.81 times, respectively. In terms of the average amount of carbon emissions from the road, railway, water transport, and aviation sectors, the average carbon emissions from 286 prefecture-level-and-above cities in China were 1,043,900 tonnes, 47,000 tonnes, 80,700 tonnes, and 69,000 tonnes for road, railway, water transport, and aviation in 2005, respectively; the average carbon emissions from road, railway, water transport, and aviation were 2,363,500 tonnes, 267,700 tonnes million tonnes, 143,300 tonnes, and 193,800 tonnes in 2015, respectively. The average carbon emissions of these four sectors increased by 1,192,700 tonnes, −20,300 tonnes, 62,600 tonnes, and 124,800 tonnes, respectively.

Looking at the total carbon emissions of China’s four major regions, the total carbon emissions of urban roads, railways, water transport, and aviation in the northeast expanded by 1.71, 0.40, 3.34, and 2.48 times, respectively. The total carbon emissions of urban roads, railways, water transport, and aviation in the eastern region expanded by 1.84 times, 0.61 times, 1.43 times, and 2.68 times, respectively. The total carbon emissions of urban roads, railways, water transport, and aviation in the central region increased by 2.80 times, 0.53 times, 4.65 times, and 3.20 times, respectively. The total carbon emissions of urban roads, railways, water transport, and aviation in the western region expanded by 2.76 times, 0.65 times, 2.41 times, and 3.31 times, respectively.

In terms of the average amount of carbon emissions in China’s four major regions, the average carbon emissions from roads in northeast, east, central and west China were 1.138 million tonnes, 48.1 million tonnes, 16.3 million tonnes, and 22.7 million tonnes in 2005, respectively, and the average carbon emissions of the agricultural sector in northeast, east, central and west China were 1.9442 million tonnes, 19.3 million tonnes, 54.4 million tonnes, and 56.2 million tonnes in 2015. From 2005 to 2015, the average values of carbon emissions of road, water transport, and aviation in the four major regions of China all increased significantly, and only the average value of carbon emissions from railways decreased significantly (Table 5).

#### 3.2.4. Capita Carbon Emission and Per Land Area Emissions

Both, in terms of carbon emissions per capita and carbon emissions per land area, China’s carbon emissions have shown a rapid increase from 2005 to 2015. The carbon emissions per capita in China’s 286 prefecture-level-and-above cities were 6.82 tonnes per person in 2005, increasing to 11.59 tonnes per person in 2015, an expansion of 1.79 times, compared to the carbon emissions per capita in China’s 286 prefecture-level-and-above cities in 2005. The carbon emissions per capita in China’s 286 prefecture-level-and-above cities were 2737.03 tonnes/km^2^ in 2005, increasing to 4613.11 tonnes/km^2^, an expansion of 1.69 times.

Looking at the average carbon emissions of China’s four major regions, from 2005 to 2015, carbon emissions per capita increased the most in the western region (expanding by 1.83 times), while carbon emissions per capita increased the least in the eastern region (expanding by 1.56 times). The eastern region had the largest ground average carbon emission intensity, with its average value increasing from 4523.48 tonnes/km^2^ in 2005 to 7407.01 tonnes/km^2^ in 2015. The western region had the smallest ground average carbon emission intensity (2521.93 tonnes/km^2^ in 2015); in terms of carbon emission increase, from 2005 to 2015, the northeast, eastern, central eastern, and western regions expanded by 1.68 times, 1.64 times, 1.72 times, and 1.78 times, respectively (Table 6).

#### 3.2.5. Direct and Indirect Carbon Emission

From 2005 to 2015, both direct and indirect carbon emissions in China showed a significant increase, with indirect carbon emissions growing significantly faster than direct carbon emissions. In terms of total emissions volume, from 2005 to 2015, the total direct carbon emissions of 286 prefecture-level-and-above cities in China increased 1.64 times, from 624,769.85 million tonnes to 1,027,499.3 million tonnes. Total indirect carbon emissions increased 2.74 times, from 386,745,800 tonnes to 106,069,600 tonnes. In terms of average volume, from 2005 to 2015, the average direct carbon emissions of 286 prefecture-level-and-above cities in China increased from 21,845,100 tonnes to 35,795,500 tonnes, an increase of 13,950,400 tonnes. The average indirect carbon emissions increased from 1,352,300 tonnes to 3,708,700 tonnes, an increase of 2,356,500 tonnes.

In terms of direct carbon emissions in the four regions, from 2005 to 2015, direct carbon emissions in cities in the northeast, east, central, and west regions increased by 1.50 times, 1.50 times, 1.77 times and 1.86 times, respectively. In terms of indirect carbon emissions from the four regions, from 2005 to 2015, indirect carbon emissions from cities in the northeast, east, central, and west regions increased by 1.3558 million tonnes, 43.6385 million tonnes, 11.243 million tonnes, and 123.252 million tonnes, respectively. Looking at the average carbon emissions of the four major regions in China, the eastern region had the highest average direct carbon emissions, while the western region had the lowest average direct carbon emissions. The central region had the lowest average indirect carbon emissions, while the eastern region still had the highest average indirect carbon emissions (Table 7).

#### 3.2.6. Carbon Emissions per GDP and Carbon Productivity

Carbon emission per unit GDP reflects carbon emission intensity, and carbon productivity reflects the opposite indicator, i.e., the larger the carbon emission per unit GDP, the higher the carbon emission intensity and the lower the carbon productivity. The smaller the carbon emission per unit GDP, and the higher the carbon productivity.

In 2005, the average carbon emission per unit GDP in 286 prefecture-level-and-above cities in China was 4.68 tonnes/yuan, which dropped to 2.37 tonnes/yuan in 2015, a decrease of 49.32%. In 2005, the average carbon emission per unit GDP in the primary, secondary and tertiary industries in Chinese cities was 0.38 tonnes/yuan, 8.62 tonnes/yuan, and 0.12 tonnes/yuan. In 2015, the average carbon emission per unit GDP in the primary, secondary, and tertiary industries was 0.38 tonnes/yuan, 8.62 tonnes/yuan, and 0.12 tonnes/yuan, respectively (Table 8).

**Figure 3:**
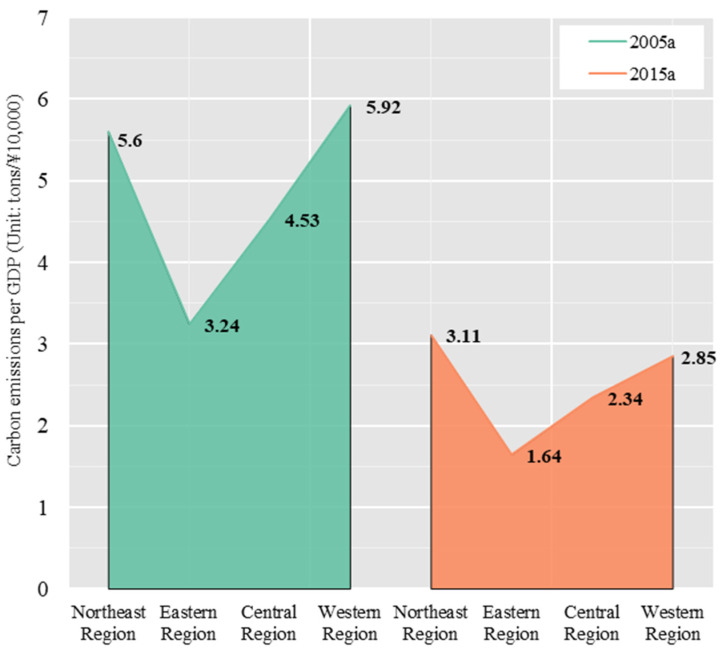
Carbon emissions per GDP in four major regions of China.

Looking at the four major regions, from 2005 to 2015, the average carbon emissions of the primary and secondary industries in the northeastern, eastern, central, and western regions of China all declined significantly, while the average carbon emissions of the tertiary industry increased (Figure 3).

In terms of the total and average carbon emissions by sector from 2005–2015, in China’s 286 cities they are expanding, but surprisingly, in terms of carbon emissions per unit GDP and average carbon emissions per unit GDP from primary, secondary, and tertiary industries, the overall carbon emission intensity of China’s 286 cities decreased, implying that China has taken steps towards low carbon emission reduction and put measures into practice.

**Figure 4:**
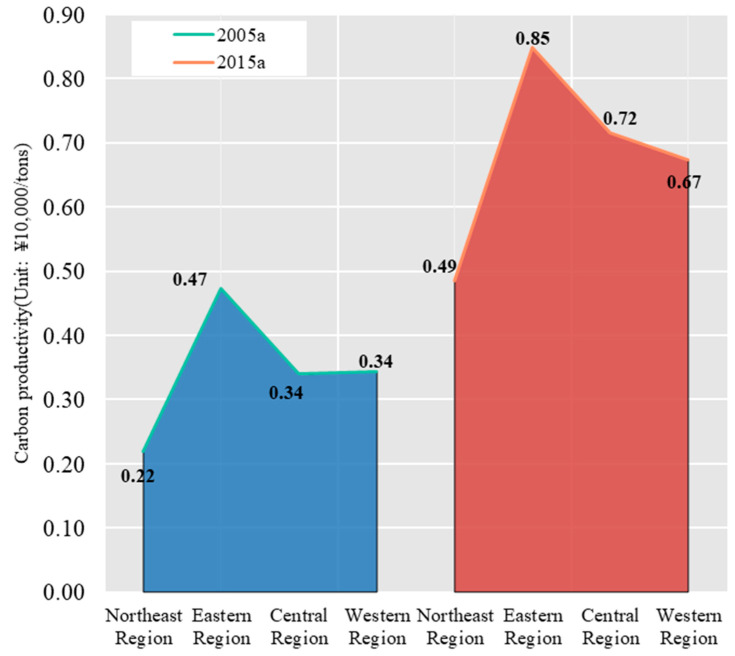
Carbon productivity in four major regions of China.

Carbon productivity is the opposite indicator of carbon emissions per unit GDP. The higher the carbon productivity, the better the effect of low-carbon emission reduction. Whether looking at the 286 cities in general or the four major regions of China, China’s carbon productivity generally showed an increasing trend from 2005 to 2015, indicating that China’s carbon emission reduction efforts achieved some success, and the change in this indicator is verified with the change in carbon emissions per unit of GDP and the change in average carbon emissions per unit GDP in the primary, secondary, and tertiary industries. Specifically, carbon productivity in China’s 286 prefecture-level and above cities increased 1.95 times, from 0.37 million yuan/tonnes in 2005 to 0.72 million yuan/tonnes in 2015. From 2005 to 2015, carbon productivity increased faster in four major regions in China, from 0.22 to 0.49 million yuan/tonnes in the northeast, from 0.47 to 0.85 million yuan/tonnes in the east, from 0.34 to 0.72 million yuan/tonnes in the central region, and from 0.34 to 0.67 million yuan/tonnes in the west (Figure 4).

## 4. Analysis of the Influencing Factors of Carbon Emissions

### 4.1. Correlation Analysis

**Figure 5:**
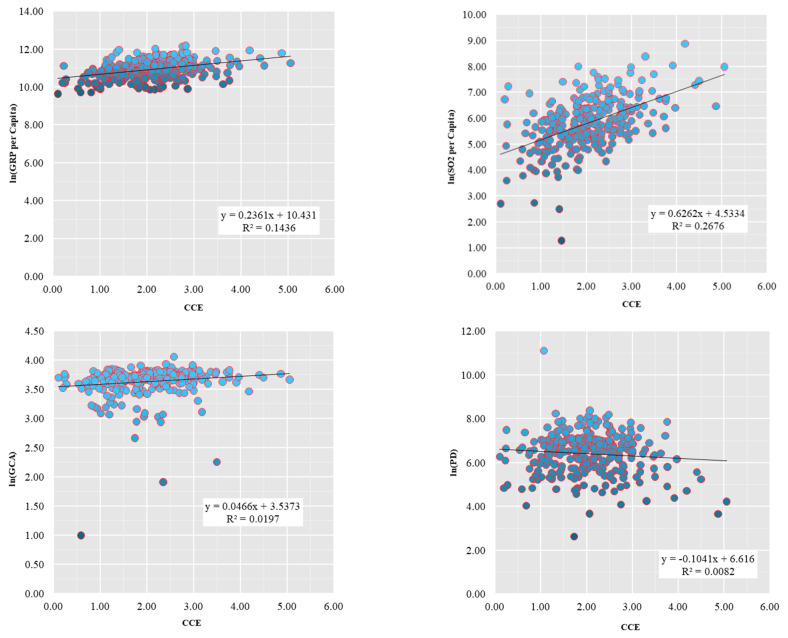

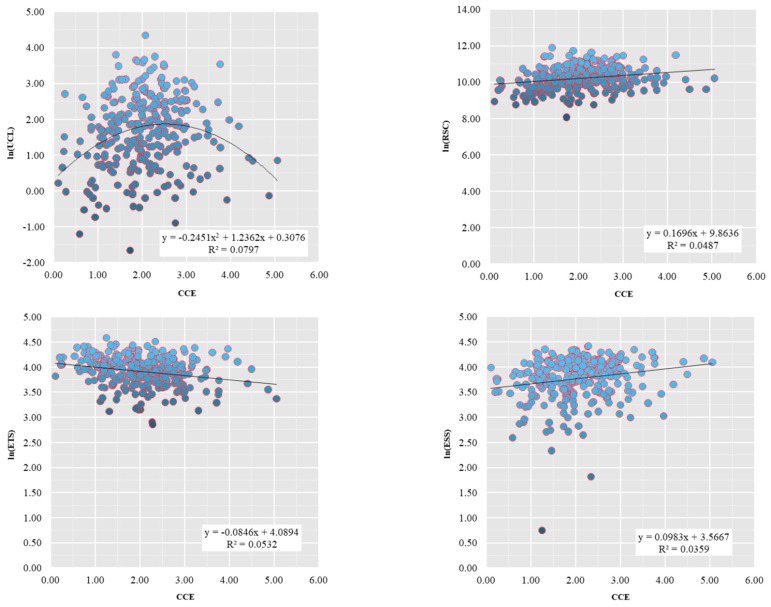
Correlation analysis between carbon emission variables.

To explore the relationship between the carbon emissions per capita and the economic, social, and ecological variables, we selected eight variables for analysis, which are GRP per capita (GRP), industrial sulfur dioxide emission per capita (SO_2_), greening coverage of built-up areas (GCA), population density (PD), proportion of urban construction land to urban area (UCL), proportion of employees in secondary sector (ESS), proportion of employees in tertiary sector (ETS), and retail sales of social consumer goods per capita (RSS) (Figure 5).

The results of the Pearson correlation analysis show that (1) carbon emissions per capita (CCE) are positively correlated with GRP per capita (GRP), industrial SO_2_ emissions per capita (SO_2_), and the proportion of employees in the secondary sector (ESS); (2) carbon emissions per capita (CCE) and population density (PD) are negatively correlated with the proportion of employees in the tertiary sector (ETS); (3) carbon emissions per capita (CCE) are (weakly) correlated with the greening coverage of built-up areas (GCA) and the proportion of urban construction land to urban area (UCL), in an inverted U-shaped curve.

In terms of the correlation between industrial structure and carbon emissions, Li studied the correlation between carbon emission intensity and the primary, secondary, and tertiary sectors in China, and concluded that the secondary sector is the main factor influencing regional carbon emission intensity, that the secondary sector is not the absolute factor influencing the increase of regional carbon emissions, and that the tertiary sector does not have a significant effect on the reduction of regional carbon emission intensity [80]. In terms of the correlation between demographic factors and carbon emissions, both ageing and urbanization had an inverted ‘U’ shaped relationship with carbon emissions [81]. In terms of the correlation between urbanization and carbon emissions, we examined the relationship between urbanization and carbon emissions in 16 emerging countries and found that the relationship between urbanization and carbon emissions in emerging developing countries was mainly positive [82]. Comparing this with our correlation analysis, we found that the influencing factor of carbon emission per capita is a multidimensional issue, not only related to economic development, development stage, and development level, but also to factors such as population density and urban construction.

### 4.2. Principal Component Analysis (PCA)

The descriptive statistics of green environment, infrastructure development, features of the built-up area, resident employment, resident savings, and consumption are shown in Table 9. Using SPSS statistical analysis software, a principal component analysis was performed on 23 independent variables, to identify their key factors (Table 10).

First, a KMO test and Bartlett test were performed on the data matrix of 286 × 23 variables, and the results showed that the Kaiser–Meyer–Olkin (KMO) measure of sampling adequacy was 0.835. According to the criterion of being able to perform principal component analysis with a KMO value greater than 0.8 proposed by statistician Kasier, the data matrix variables used in this study were able to perform a principal component analysis. Meanwhile, the significance level of Bartlett’s sphericity test was 0.001, and the principal component analysis was significant.

Second, the explained total variance was measured. The principal component analysis method was chosen in the factor analysis, based on the eigenvalues, and variables with eigenvalues greater than 1 were extracted to give the explained total variance table. In the explained total variance table, the cumulative variance contribution of the top six components reached 79.55%, which means that these six principal factors can represent 79.55% of the information of 23 variables. The six principal factors have the ability to express the information of the key factors.

Third, naming of the principal component variables. Combined with the rotated component matrix table, the main influencing factors of the six principal components were comprehensively assessed, based on the correlation coefficients in the correlation matrix. The RMB savings deposits per resident (SDR) and capita retail sales of consumer goods (RSC) in the first principal component have relatively high loadings; therefore, the first principal component can be named as ‘resident savings and consumption factor’. The three variables of industrial wastewater discharge per capita, SO_2_ per capita, and industrial smoke (dust) emissions per capita in the second principal component have relatively high loadings; therefore, the second principal component can be named as ‘pollution emission factor’. The two variables share of secondary sector in GRP and share of tertiary sector in GRP have relatively high loadings; therefore, the third principal component can be named as ‘economic structure factor’. The fourth principal component, population density (PD), has a relatively high loading, so can be named as ‘population density factor’. The GRP growth rate in the fifth principal component has a high loading, so it can be named as the ‘economic growth factor’. The green covered area of built-up area (GCA) in the sixth principal component can be named as the ‘resident employment factor’ (Table 11).

Finally, the scores of the six main factors were measured using SPSS software, and six factor scores for principal component analysis were obtained: factor score 1, factor score 2, factor score 3, factor score 4, factor score 5, and factor score 6.

### 4.3. Stepwise Regression Analysis

A carbon emission regression model was constructed with carbon emission per capita as the dependent variable and the six variables of the main factor analysis as the independent variables.

$$Y=\beta_{0}+\alpha Factor1+\beta Factor2+\chi Factor3+\delta Factor4+jFactor5+\varphi Factor6+\varepsilon_{i}$$

β_0_ is the constant term, the coefficients α~ψ are the coefficients to be determined, the coefficients to be determined can be derived by multiple regression analysis, and ε_i_ is the error term.

The first to the sixth principal components are the resident savings and consumption factor, pollution emission factor, economic structure factor, population density factor, economic growth factor, and resident employment factor, respectively. We expect that the residential savings and consumption factor, pollution emission factor, population density factor, economic growth factor, and residential employment factor may all bring about an increase in carbon emissions, while the economic structure factor may bring about an increase or a decrease in carbon emissions.

The basic idea of the stepwise regression analysis method is to automatically select the most important variables from the large number of available variables and build a predictive or explanatory model for regression analysis. The independent variables are introduced one by one, and their partial regression sum of squares is tested for significance. At the same time, after each new independent variable is introduced, the old independent variables are tested one by one, and the independent variables with insignificant regression sums of squares are eliminated. In this way, we keep eliminating while introducing, until no new variables are introduced and no old variables are removed. The essence is to establish the ‘optimal’ multiple linear regression equation (Table 12). Our regression model explains the contribution of the per capita carbon emissions impact factor with an adjusted R-squared of 0.400, meaning that the factors in the model explain 40% of the impact factor in per capita carbon emissions; leaving 60% of the impact factor as not yet included in this model.

Stepwise regression was used to screen and remove variables that cause multi-collinearity in the following steps: a simple regression is first done with the explanatory variables for each of the explanatory variables considered, and then the regression equation corresponding to the explanatory variable that contributes most to the explanatory variable is used as the basis for gradually introducing the remaining explanatory variables. After the stepwise regression is performed, so that the final explanatory variables retained in the model are both significant and free from severe multi-collinearity. The results of the stepwise regression analysis showed that factor 6 was excluded due to the problem of cointegration, and the final regression coefficients were as in Table 7.

Based on the unstandardized regression coefficients in Table 13, we constructed the carbon emission regression model.

Y=0.346Factor1+0.239Factor2+0.159Factor3−0.156Factor4−0.228Factor5+2.051

Factor 1: resident savings and consumption showed a positive relationship with carbon emissions per capita, with a t-value of 9.201 and a *p*-value less than 0.01, indicating that the Factor1 variable in the regression model is significant. The regression coefficient is 0.346, and each 1% increase in residents’ savings and consumption will bring a positive increase of 0.346% in per capita carbon emissions, controlling other variables as constant.

Factor 2: Pollution emission factor had a positive effect on carbon emissions per capita, with a t-value of 6.347 and a *p*-value less than 0.01, indicating that pollution emissions can significantly promote carbon emissions. The regression coefficient is 0.239, and every 1% increase in pollution emission factor will bring a positive increase of 0.239% in per capita carbon emissions, keeping other variables constant.

Factor 3: The relationship between the economic structure factor and per capita carbon emission is complex, and our study used the number of employees in the three industries to represent the economic structure factor. In the correlation analysis, the proportion of employees in the secondary sector (ESS) was positively correlated with carbon emissions per capita, while the proportion of employees in tertiary sector (ETS) was negatively correlated with carbon emissions per capita. In the regression analysis, there was a positive correlation between the economic structure factor and per capita carbon emissions, which was due to the fact that the current energy structure of China is still dominated by coal and supplemented by clean energy. Despite the Chinese government’s efforts to reduce energy consumption and improve energy conservation and emission reductions, it has not yet fundamentally changed the energy structure, which is the key factor leading to the positive correlation between the economic structure factor and carbon emission per capita. The Factor3 variable in the regression model was significant, with a regression coefficient of 0.159, indicating that every 1% adjustment in economic structure will bring a 0.159% increase in carbon emissions per capita.

Factor 4: Population density and carbon emissions per capita showed a negative relationship, with a t-value of −4.141 and a *p*-value less than 0.01, indicating that the Factor4 variable in the regression model was significant. The regression coefficient is −0.156, which shows that every 1% increase in population density will lead to a 0.156% decrease in per capita carbon emissions, controlling for other variables. The rapid urbanization process, the concentration of population toward towns and cities, the spatial clustering of populations, and the development of urban agglomerations mitigate carbon emissions per capita.

Factor 5: The economic growth factor and carbon emissions per capita showed a negative relationship, with a t-value of 9.201 and a *p*-value less than 0.01, indicating that the Factor5 variable in the regression model is significant. The regression coefficient is 0.228, and every 1% increase in economic growth factor will bring a negative increase of 0.228% in per capita carbon emissions, keeping other variables constant.

## 5. Discussion

This study empirically analyzed the spatial heterogeneity of carbon emissions in 286 cities at prefecture level and above in China, analyzed the factors for the evolution of regional differences in carbon emissions in the four major regions of China, revealed the factors influencing carbon emissions in Chinese cities, and provided an empirical basis for the formulation of low-carbon emission reduction measures and policies in China. This study is different from those of other scholars, such as Liu and Li, who analyzed the temporal and spatial pattern of China’s carbon emissions [83,84]. We conducted a spatial heterogeneity analysis of carbon emissions in 20 sectors of 286 prefecture-level-and-above cities in China, and the study was able to observe the spatio-temporal differences of carbon emissions in China from a multi-sectoral perspective. In terms of the analysis of the influencing factors of carbon emissions, Xu revealed the existence of an environmental Kuznets curve for carbon emissions at the provincial level in China [85], but an analysis of influencing factors is still lacking. Although scholars such as Zhao, Cai, and Zhao [86,87,88] analyzed the influencing factors of carbon emissions, some of their analyses were spatially scaled to Chinese provinces, while the analysis of specific factors only focused on aspects such as urbanization and industrial composition, lacking an analysis of the influence of demographic, economic, and environmental variables. Our study has taken into account these factors, which will contribute to the study of the impact mechanism of low carbon emission reductions in China. Of course, our study also has certain shortcomings, as policy factor variables are missing from our explanatory variables, due to the limited availability of data. In China, policy factors have a strong influence on economic development, social transformation, and low carbon emission reduction. To fill this gap, we included policy factors in the policy recommendation section.

## 6. Conclusions

Using a combination of comparative analysis, regional difference analysis, correlation analysis, principal component analysis, and stepwise regression analysis, the spatial heterogeneity of carbon emissions and its influencing factors in 286 prefecture-level-and above-cities in China were studied, and the following conclusions were drawn:

(1) In the analysis of the spatial heterogeneity of carbon emissions in 286 cities at prefectural level and above in China, the regional differences in six sectors, including urban household carbon emissions, railway carbon emissions, aviation carbon emissions, carbon emissions per unit GDP, carbon emissions per unit GDP in primary industry, and carbon emissions per unit GDP in secondary industry all widened, while the regional differences in rural household carbon emissions, agricultural carbon emissions, industrial carbon emissions, service carbon emissions, road carbon emissions carbon emissions, carbon emissions from water navigation, carbon emissions from transportation, direct carbon emissions, indirect carbon emissions, total carbon emissions, carbon emissions per capita, carbon emissions per unit of land area, carbon emissions per unit of GDP in the tertiary sector, and carbon productivity all narrowed; the regional differences narrowed in 14 sectors.

(2) In terms of the spatial heterogeneity of carbon emissions in the four major regions of China, including the northeast, eastern, central, and western regions, the intra-group differences in six sectors, including urban household carbon emissions, railway carbon emissions, aviation carbon emissions, carbon emissions per unit GDP, carbon emissions per unit GDP in the primary industry, and carbon emissions per unit GDP in the secondary industry, are significant, as shown in the northeast, eastern, central, and western regions. The within-group differences in carbon emissions of the six sectors in China are different.

(3) From 2005 to 2015, the carbon emissions of rural households in China were higher than those of urban households, and the carbon emissions of the three major sectors, namely agriculture, industry, and services, increased significantly. The carbon emissions from the four major sectors of ‘iron, public, and infrastructure’ are increasing at a significant rate, and carbon emissions per capita and per land area are also increasing, with indirect carbon emissions increasing at a significantly faster rate than direct carbon emissions. Although the carbon emissions of all sectors in China’s 286 cities are expanding, it is surprising to see that the overall carbon emission intensity of China’s 286 cities has decreased, in terms of carbon emissions per unit GDP and the average carbon emissions per unit GDP of primary, secondary and tertiary industries, which means that China has taken steps to reduce carbon emissions and put them into practice.

(4) From the results of the correlation analysis, carbon emissions per capita (CCE) are positively correlated with GRP per capita (GRP), industrial SO_2_ emissions per capita (SO_2_), and the proportion of employees in the secondary industry (ESS). Carbon emissions per capita (CCE) and population density (PD) are negatively correlated with the proportion of workers in the tertiary sector (ETS). Carbon emission per capita (CCE) is (weakly) correlated with the green coverage of built-up areas (GCA) and the proportion of urban construction land to urban area (UCL), in an inverted U-shaped curve.

(5) In terms of the explanatory factors of carbon emissions, residential savings and consumption are positively correlated with per capita carbon emissions, and pollution emission factors are positively correlated with per capita carbon emissions, with each 1% increase in pollution emission factors bringing a 0239% positive increase in per capita carbon emissions; the regression coefficient is 0.159, indicating that each 1% adjustment in economic structure will bring a 0.159% increase in per capita carbon emissions. Every 1% increase in population density will lead to a 0.156% negative increase in per capita carbon emissions; and every 1% increase in the economic growth factor will lead to a 0.228% negative increase in per capita carbon emissions, controlling for other variables.

## 7. Policy Suggestions

In October 2021, the northeast region of China was the first to start limiting electricity. Subsequently, Guangdong, Anhui, Jiangsu, and Zhejiang started to restrict electricity, and some high energy-consuming enterprises restricted electricity for 10 days. The apparent reason for this is the rising price of coal; the essential reason is the lack of capacity needed for the low-carbon transition.

China’s power restriction is an inevitable event, and the root problem behind the power restriction is long-term and global. The issue of coal utilization is a paradoxical problem that China has to face in the long run. According to our empirical study, the pollution emission factor has a significantly positive correlation with carbon emission per capita, and pollution emission is mostly dominated by high energy-consuming enterprises. The empirical study also showed that economic restructuring still exacerbates the increase of carbon emission per capita, indicating that a fundamental transformation of economic structure has not yet occurred. Therefore, we suggest the following:

First, strengthen the coordination of economic development and energy structure, to balance energy supply and demand, matching construction. After 2000, China’s energy demand exceeded expectations, leading to a shortage of energy supply and a nationwide short supply of coal, electricity, and oil, which stimulated energy production capacity. After the 2008 financial crisis, energy supply slowed down, but the Chinese government’s ‘four trillion’ infrastructure construction plan in the process of economic recovery stimulated building capacity and exacerbated the tight energy supply situation. In 2021, the energy supply is again tight and power supply is restricted, indicating the incongruity between China’s energy supply and economic development. Therefore, properly handling the contradiction between the transformation of energy structure and the transformation of economic development is the necessary path for China to follow a sustainable development path and achieve energy self-sufficiency in the future.

Second, accelerate the construction of new infrastructure capacity to match the new energy system. The path to a low-carbon transition is important for every country, and for China, the world’s largest developing country, accelerating the construction of new infrastructure to match new energy construction is a hardware condition and a technical condition for guaranteeing the transformation of China’s energy structure. For example, the renovation of the distribution grid could significantly reduce energy consumption. It is recommended to strengthen the coordination and coupling of energy and infrastructure construction, accelerate the synergistic use of media and new energy, etc., build a supply system with complementary energy sources, and promote the construction of a support network system for the new energy supply system.

Third, promote green development and a green and low-carbon lifestyle. Green development is a method of economic growth and social development that aims at efficiency, harmony, and sustainability. Green lifestyle aims to make green consumption, green travel, and green living become part of people’s conscious actions by encouraging residents to use green products and encouraging people to participate in green voluntary services, as well as guiding people to establish the concept of green growth and sharing together. The former focuses on the comprehensive, coordinated, and sustainable development of the economy, society, and environment at a macro level, while the latter focuses on the transformation of residents’ personal lifestyles at a micro level, focusing on low-carbon, energy-saving, and environmental protection. Green development means promoting the transformation of the city, from the old sloppy urban economic growth mode, to an intensive economic growth mode, while green lifestyle means improving the residents’ concept of environmental protection, focusing on the recycling of resources in life, using public transport as the main mode of travel, strengthening the conscious process of waste separation, and saving water and electricity and other daily resources.

## Figures and Tables

**Table 1:** Within-group differences, between group differences, and total differences.

	Theil Index	Within-Group Differences		Between Group Differences		Total Differences	
Indicators		2005a	2015b	2005a	2015b	2005a	2015b
Urban Household		0.485	0.685	0.045	0.010	0.529	0.695
Rural Household		0.810	0.772	0.087	0.012	0.897	0.784
Agriculture		0.374	0.347	0.034	0.012	0.408	0.359
Industry		0.415	0.363	0.045	0.025	0.460	0.387
Service		0.801	0.703	0.005	0.028	0.806	0.731
Road		0.267	0.254	0.124	0.050	0.392	0.304
Railway		0.372	0.381	0.014	0.011	0.387	0.392
Waterborne Navigation		1.173	1.177	0.632	0.333	1.805	1.510
Aviation		1.778	2.161	0.415	0.365	2.193	2.526
Transportation		0.305	0.307	0.144	0.070	0.449	0.377
Direct Emission		0.366	0.316	0.047	0.025	0.412	0.340
Indirect Emission		1.495	0.951	0.251	0.218	1.746	1.169
Total Emission		0.369	0.307	0.052	0.035	0.421	0.341
Capita Carbon Emission		0.455	0.435	0.016	0.027	0.471	0.463
Per Land Area Emission		0.524	0.470	0.107	0.095	0.631	0.565
Carbon Emissions per GDP		0.261	0.304	0.028	0.027	0.289	0.331
Primary Industry		0.250	0.258	0.008	0.006	0.257	0.263
Secondary Industry		0.286	0.387	0.046	0.045	0.332	0.431
Tertiary Industry		0.474	0.152	0.122	0.047	0.595	0.200
Carbon Productivity		0.216	0.198	0.024	0.012	0.240	0.211

**Table 2:** Within group differences in four regions of China.

	Theil Index	Northeast Region		Eastern Region		Central Region		Western Region	
Indicators		2005a	2015b	2005a	2015b	2005a	2015b	2005a	2015b
Urban Household		0.040	0.035	0.207	0.229	0.053	0.057	0.185	0.363
Rural Household		0.013	0.017	0.157	0.413	0.079	0.113	0.561	0.229
Agriculture		0.026	0.044	0.104	0.090	0.062	0.080	0.183	0.133
Industry		0.025	0.020	0.210	0.127	0.071	0.082	0.109	0.133
Service		0.085	0.083	0.193	0.275	0.169	0.073	0.353	0.273
Road		0.035	0.022	0.139	0.132	0.035	0.032	0.058	0.069
Railway		0.021	0.012	0.198	0.198	0.049	0.057	0.104	0.113
Waterborne Navigation		0.019	0.116	0.950	0.702	0.050	0.163	0.154	0.197
Aviation		0.051	0.077	1.462	1.511	0.075	0.168	0.189	0.406
Transportation		0.031	0.023	0.189	0.174	0.031	0.032	0.055	0.078
Direct Emission		0.023	0.017	0.186	0.119	0.059	0.063	0.098	0.116
Indirect Emission		0.220	0.071	0.922	0.543	0.142	0.121	0.211	0.217
Total Emission		0.027	0.017	0.194	0.125	0.055	0.056	0.093	0.108
Capita Carbon Emission		0.016	0.028	0.060	0.040	0.073	0.064	0.306	0.303
Per Land Area Emission		0.025	0.032	0.229	0.177	0.152	0.152	0.118	0.110
Carbon emissions per GDP		0.013	0.041	0.046	0.038	0.059	0.088	0.142	0.137
Primary Industry		0.013	0.023	0.052	0.067	0.096	0.079	0.089	0.088
Secondary Industry		0.029	0.083	0.063	0.054	0.060	0.115	0.135	0.136
Tertiary Industry		0.063	0.054	0.016	0.032	0.122	0.025	0.273	0.042
Carbon Productivity		0.008	0.013	0.076	0.057	0.050	0.055	0.082	0.072

**Table 3:** Carbon emissions from urban and rural households.

	Carbon Emission	Urban Households (10^4^ tonnes)		Rural Households (10^4^ tonnes)	
Region		2005a	2015a	2005a	2015a
Northeast Region		45.70	29.86	20.21	33.61
Eastern Region		63.82	40.73	57.66	51.49
Central Region		32.38	29.05	53.65	56.75
Western Region		34.75	35.10	102.57	44.59

**Table 4:** Comparison of the average value of carbon emissions from the agricultural, industrial, and service sectors in the four major regions of China (unit: 10^4^ tonnes).

	Carbon Emission	Agricultural Sector		Industrial Sector		Service Industry Sector	
Region		2005a	2015a	2005a	2015a	2005a	2015a
Northeast Region		28.98	42.99	1775.30	2536.60	21.11	161.60
Eastern Region		33.80	34.84	2804.79	4172.50	20.88	98.72
Central Region		24.08	34.65	1559.78	2718.36	20.35	75.56
Western Region		17.17	26.54	1411.08	2598.15	25.42	98.21

**Table 5:** Comparison of the average value of carbon emissions from road, railway, waterborne navigation, and aviation in the four major regions of China (Unit: 10^4^ tonnes).

	Carbon Emission	Road		Railway		Waterborne Navigation		Aviation	
Region		2005a	2015a	2005a	2015a	2005a	2015a	2005a	2015a
Northeast Region		113.80	194.42	4.81	1.93	1.63	5.44	2.27	5.62
Eastern Region		181.15	332.58	4.89	2.98	22.41	31.96	16.67	44.67
Central Region		65.91	184.51	5.62	2.99	2.24	10.42	1.47	4.72
Western Region		58.26	160.69	3.60	2.35	1.46	3.52	3.86	12.79

**Table 6:** Comparison of the average value of capita carbon emission and per land area emissions in four major regions of China (Unit: t/person; t/km^2^).

	Carbon Emission	Capita Carbon Emission		Per Land Area Emissions	
Region		2005a	2015a	2005a	2015a
Northeast Region		7.65	12.84	1655.73	2774.71
Eastern Region		6.17	9.65	4523.48	7407.01
Central Region		5.45	9.05	2660.63	4577.92
Western Region		8.44	15.46	1412.97	2521.93

**Table 7:** Comparison of the average value of direct and indirect carbon emissions in four major regions of China (Unit: 10^4^ tonnes).

	Carbon Emission	Direct Emission		Indirect Emission	
Region		2005a	2015a	2005a	2015a
Northeast Region		2013.81	3012.06	205.78	209.77
Eastern Region		3206.04	4810.49	258.26	760.15
Central Region		1765.50	3117.02	39.83	180.37
Western Region		1601.58	2981.94	70.85	216.18

**Table 8:** Comparison of the carbon emissions per primary industry, secondary industry, and tertiary industry in four major regions of China (Unit: tonnes/¥10,000).

	Carbon Emission	Primary Industry		Secondary Industry		Tertiary Industry	
Region		2005a	2015a	2005a	2015a	2005a	2015a
Northeast Region		0.45	0.23	12.18	7.01	0.14	0.85
Eastern Region		0.37	0.17	5.36	2.79	0.05	0.33
Central Region		0.42	0.21	8.05	4.01	0.12	0.46
Western Region		0.32	0.18	11.09	4.82	0.20	0.58

**Table 9:** Descriptive statistics of independent variables.

Target Layer	Indicator Layer	Max	Min	AVG	STD	Unit
Economic Development	GRP per capita	19.58	1.54	6.27	3.22	10,000¥
	GRP growth rate	15.30	−9.98	7.55	3.35	%
	Share of secondary sector in GRP	74.45	14.33	46.71	11.05	%
	Share of tertiary sector in GRP	79.65	22.36	46.70	10.90	%
Industrial level	Number of industrial enterprises owned by 10,000 people	59.41	0.40	9.69	8.59	pcs
	Total industrial output per capita	156.34	0.94	27.30	24.24	10,000¥
Pollution emission	Industrial wastewater discharge per capita	359.78	0.91	58.27	57.51	t
	SO_2_ per capita	7184.07	3.58	536.87	684.03	kg
	Industrial smoke (dust) emissions per capita	16,165.72	3.14	508.15	1336.21	kg
Green Environment	Green space per capita	428.31	1.74	46.85	48.10	m^2^
	Parkland area per capita	65.95	0.83	10.53	7.43	m^2^
	Greening coverage area per capita in built-up areas (GCB)	215.67	0.68	39.05	23.10	ha
	Green covered area of built-up area (GCA)	57.94	2.71	38.82	6.93	%
Infrastructure Development	Fixed asset investment per capita (FAI)	19.96	0.93	5.73	3.36	10,000¥
	Urban road area per capita (URA)	106.27	1.25	13.19	9.53	m^2^
Features of the built-up area	Built-up area per capita (BUA)	5.55	0.25	1.04	0.64	km^2^/10,000 person
	Population density (PD)	65,911.00	13.85	1068.63	3892.50	person/km^2^
	Urban construction land as proportion of urban area (UCL)	77.32	0.19	8.40	9.08	%
Resident employment	Number of employees in urban units with 10,000 people	13,386.64	125.56	2200.93	1488.35	person
	Proportion of employees in secondary sector (ESS)	82.60	2.11	46.46	15.35	%
	Proportion of employees in tertiary sector (ETS)	97.89	17.38	52.50	14.91	%
Resident savings and consumption	RMB savings deposits per resident (SDR)	28.17	1.13	5.86	3.57	10,000¥
	Capita retail sales of consumers goods (RSC)	14.60	0.32	3.32	2.19	10,000¥

**Table 10:** Total explained variance.

Ingredients	Initial Eigenvalue			Extraction of Squares and Loading		
	Total	Variance(%)	Cumulative(%)	Total	Variance(%)	Cumulative(%)
1	8.83	38.39	38.39	8.83	38.39	38.39
2	3.02	13.12	51.50	3.02	13.12	51.50
3	2.56	11.13	62.63	2.56	11.13	62.63
4	1.53	6.64	69.27	1.53	6.64	69.27
5	1.35	5.87	75.14	1.35	5.87	75.14
6	1.02	4.41	79.55	1.02	4.41	79.55

Extraction method: principal component analysis.

**Table 11:** Rotated Component Matrix.

Variables	1	2	3	4	5	6
GRP per capita	0.77	−0.02	0.30	0.12	0.27	−0.03
GRP growth rate	−0.01	−0.16	−0.03	0.05	0.83	−0.07
Share of secondary sector in GRP	0.02	0.18	0.82	−0.01	0.25	0.26
Share of tertiary sector in GRP	0.34	−0.07	−0.72	0.31	−0.28	−0.19
Number of industrial enterprises owned by 10,000 people	0.22	0.57	0.16	0.41	0.40	0.15
Total industrial output per capita	0.44	0.53	0.31	0.35	0.41	0.11
Industrial wastewater discharge per capita	0.21	0.77	0.09	0.14	0.14	0.07
SO_2_ per capita	0.13	0.87	0.04	−0.15	−0.19	−0.04
Industrial smoke (dust) emissions per capita	0.09	0.80	−0.03	−0.10	−0.20	−0.06
Green space per capita	0.81	0.15	0.00	0.01	0.03	0.35
Parkland area per capita	0.78	0.22	−0.02	0.13	−0.03	0.20
Greening coverage area per capita in built-up areas (GCB)	0.81	0.27	0.06	0.02	−0.07	0.41
Green covered area of built-up area (GCA)	0.24	−0.04	0.19	0.17	−0.08	0.84
Fixed asset investment per capita (FAI)	0.69	0.08	0.17	0.13	0.43	−0.09
Urban road area per capita (URA)	0.75	0.31	0.05	0.01	0.06	0.21
Built-up area per capita (BUA)	0.84	0.27	−0.08	−0.05	−0.07	−0.04
Population density (PD)	0.08	−0.05	0.15	0.90	0.09	0.11
Urban construction land as proportion of urban area (UCL)	0.48	0.05	0.12	0.77	0.02	0.08
Number of employees in urban units with 10,000 people	0.81	−0.01	0.22	0.27	−0.10	−0.08
Proportion of employees in secondary sector (ESS)	0.15	0.01	0.84	0.32	−0.11	0.01
Proportion of employees in tertiary sector (ETS)	−0.22	0.02	−0.82	−0.22	0.20	0.08
RMB savings deposits per resident (SDR)	0.85	0.04	−0.10	0.29	−0.10	−0.03
Capita retail sales of consumers goods (RSC)	0.80	−0.03	0.01	0.44	0.09	−0.03

Extraction method: principal component analysis.

**Table 12:** Models Summary.

Model	R	R-Square	Adjusted R-Square	Standard Error in Estimation
1	0.420 ^a^	0.176	0.174	0.749
2	0.510 ^b^	0.260	0.255	0.711
3	0.581 ^c^	0.337	0.330	0.675
4	0.612 ^d^	0.374	0.366	0.657
5	0.640 ^e^	0.410	0.400	0.639

^a^. Predictive variables: (Constant), REGR factor score 1 for analysis 1. ^b^. Predictive variables: (Constant), REGR factor score 1 for analysis 1, REGR factor score 2 for analysis 1. ^c^. Predictive variables: (Constant), REGR factor score 1 for analysis 1, REGR factor score 2 for analysis 1, REGR factor score 5 for analysis 1. ^d^. Predictive variables: (Constant), REGR factor score 1 for analysis 1, REGR factor score 2 for analysis 1, REGR factor score 5 for analysis 1, REGR factor score 3 for analysis 1. ^e^. Predictive variables: (Constant), REGR factor score 1 for analysis 1, REGR factor score 2 for analysis 1, REGR factor score 5 for analysis 1, REGR factor score 3 for analysis 1, REGR factor score 4 for analysis 1.

**Table 13:** Regression coefficients ^a^.

Model	Non-Standardized Coefficients	Standard Errors	Standard Coefficients	t	Sig.
(Constant)	2.051	0.038		54.584	0.000
Factor1	0.346	0.038	0.42	9.201	0.000
Factor2	0.239	0.038	0.29	6.347	0.000
Factor3	0.159	0.038	0.193	4.22	0.000
Factor4	−0.156	0.038	−0.189	−4.141	0.000
Factor5	−0.228	0.038	−0.277	−6.068	0.000

^a^. Dependent variable: Capita carbon emissions.

## Data Availability

China City Greenhouse Gases Emission Dataset (2005) and China City Greenhouse Gases Emission Dataset (2015), China Urban Statistical Yearbook 2016 and China Urban Construction Statistical Yearbook 2015.

## Abbreviations

The following abbreviations are used in this manuscript: CCE: Capita Carbon Emissions; TCE: Total Carbon Emissions; SO_2_ per Capita: Capita Volume of Sulphur Dioxide Emission; GCA: Green Covered Area as Proportion of Completed Area; PD: Population Density; UCL: Urban Built-Up Area as Proportion of Urban Area; ESS: Proportion of Employees in the Secondary Sector; ETS: Proportion of Employees in the Tertiary Sector; RSC: Capita Retail Sales of Consumer Goods.
